# Diagnosis of erectile dysfunction can be used to improve screening for Type 2 diabetes mellitus

**DOI:** 10.1111/dme.13783

**Published:** 2018-08-02

**Authors:** R. M. Carrillo‐Larco, A. C. Luza‐Dueñas, M. Urdániga‐Hung, A. Bernabé‐Ortiz

**Affiliations:** ^1^ CRONICAS Centre of Excellence in Chronic Diseases Universidad Peruana Cayetano Heredia Lima Peru; ^2^ Department of Epidemiology and Biostatistics School of Public Health Imperial College London London UK; ^3^ School of Medicine Faculty of Health Sciences Universidad Peruana de Ciencias Aplicadas – UPC Lima Peru; ^4^ Faculty of Epidemiology and Population Health London School of Hygiene and Tropical Medicine London UK

## Abstract

**Aims:**

To assess the diagnostic accuracy of four undiagnosed Type 2 diabetes mellitus risk scores accounting for erectile dysfunction status.

**Methods:**

This was a population‐based cross‐sectional study. Type 2 diabetes was defined according to a oral glucose tolerance test and self‐reported physician diagnosis. Erectile dysfunction was defined according to the answer to the question, ‘Have you had difficulties obtaining an erection in the last 6 months?’ (yes/no). The risk scores used were the FINDRISC, LA‐FINDRISC, American Diabetes Association score and the Peruvian Risk Score. A Poisson regression model was fitted to assess the association between Type 2 diabetes and erectile dysfunction. The area under the receiver‐operating characteristic curve was estimated overall and by erectile dysfunction status.

**Results:**

A total of 799 men with a mean (sd) age of 48.6 (10.7) years were included in the study. The overall prevalence of Type 2 diabetes was 9.3%. Compared with healthy men, men with Type 2 diabetes had 2.71 (95% CI 1.57–4.66) higher chances of having erectile dysfunction. Having excluded men aware of Type 2 diabetes status (*N*=38), the area under the receiver‐operating characteristic curve of three of the risk scores (not the American Diabetes Association score) improved among those who had erectile dysfunction in comparison with those who did not; for example, the area under the receiver‐operating characteristic curve of the LA‐FINDRISC score was 89.6 (95% CI 78.7–99.9) in men with erectile dysfunction and 76.5 (95% CI 68.5–84.4) overall.

**Conclusions:**

In a population‐based study, erectile dysfunction was more common in men with Type 2 diabetes than in the otherwise healthy men. Screening for erectile dysfunction before screening for Type 2 diabetes seems to improve the accuracy of well‐known risk scores for undiagnosed Type 2 diabetes.

## What's new?

- Erectile dysfunction is associated with diabetes; however, how to use it to identify diabetes cases has not been studied. The aim of this study was to determine whether the diagnostic accuracy of four well‐known risk scores for undiagnosed diabetes improved in men with erectile dysfunction, in comparison to men without this comorbidity.
- Most of the assessed risk scores showed a better capacity to distinguish between a man with diabetes and a healthy man when applied to men with erectile dysfunction.
- These findings, although they need to be verified by more comprehensive studies, suggest that erectile dysfunction ascertainment could improve diabetes screening.

**What's new?**

- Erectile dysfunction is associated with diabetes; however, how to use it to identify diabetes cases has not been studied. The aim of this study was to determine whether the diagnostic accuracy of four well‐known risk scores for undiagnosed diabetes improved in men with erectile dysfunction, in comparison to men without this comorbidity.
- Most of the assessed risk scores showed a better capacity to distinguish between a man with diabetes and a healthy man when applied to men with erectile dysfunction.
- These findings, although they need to be verified by more comprehensive studies, suggest that erectile dysfunction ascertainment could improve diabetes screening.

## Introduction

The prevalence of Type 2 diabetes has increased globally in recent decades and has increased faster in low‐ and middle‐income countries where screening, diagnostic and treatment resources are scarce 1. Furthermore, costs associated with Type 2 diabetes are likely to increase even if its prevalence decreases 2, which would make it harder for low‐ and middle‐income countries to secure diabetes care for those diagnosed with Type 2 diabetes and for people at high risk of the disease. In this context, finding new methods or approaches to improve the identification of high‐risk individuals is increasingly important. Studying factors associated with Type 2 diabetes and risk factors is the basis for identifying the characteristics of those people who would most benefit from Type 2 diabetes screening.

Type 2 diabetes has been extensively associated with erectile dysfunction (ED), with a global prevalence of 50% in men with Type 2 diabetes 3; however, there have been few studies on how this seemingly important associated factor can be used to improve Type 2 diabetes screening. The aim of the present study, therefore, was to determine whether the diagnostic accuracy of risk scores for undiagnosed Type 2 diabetes (e.g. FINDRISC 4) improves according to ED status in a population‐based sample in Peru. The hypothesis tested was that a risk score for undiagnosed Type 2 diabetes would discriminate better had we known that ED was present. This would further support ED screening in middle‐aged men as a first approach to improving the chances of successfully diagnosing Type 2 diabetes. This approach would be particularly relevant in resource‐limited settings where more expensive or even unavailable diagnostic produces need to be used wisely.

## Participants and methods

### Study design and setting

This was a cross‐sectional analysis of a population‐based study conducted in Tumbes, northern Peru. Tumbes has a population of 240 590 people (in 2016), of whom at least 10% are considered poor, and the overall life expectancy is 74.7 years 5. Notably, the prevalence of Type 2 diabetes in Tumbes exceeds the national average 6.

### Study population

Using a recent census of the study area, participants were selected using a sex‐stratified single‐stage random sampling method. Men aged 30–69 years, capable of giving informed consent and without physical disabilities preventing them from anthropometric evaluation, were eligible for the present study. One individual per household was included.

### Variables

The outcome of interest was Type 2 diabetes, defined according to self‐reported physician diagnosis or oral glucose tolerance test criteria: fasting glucose level ≥7.0 mmol/l (≥126 mg/dl) or 2‐h plasma glucose ≥11.1 mmol/l (≥200 mg/dl) 7. The Cobas Modular Platform automated analyser with Roche Diagnostics reagents was used.

The exposure of interest was ED, defined as a positive answer to the question: ‘During the past 6 months, have you had difficulties obtaining an erection?’. This question was based on the Survey of Autonomic Symptoms in people with Type 2 diabetes 8.

Four risk scores for undiagnosed Type 2 diabetes were used: FINDRISC 4, LA‐FINDRISC 9, the American Diabetes Association (ADA) score 7, and the Peruvian Risk Score 10. Variables to inform these risk scores were assessed using questionnaires (e.g. physical activity) or anthropometric assessment (e.g. waist circumference, weight and height).

Other collected variables included: an assets index based on facilities and goods owned by the household (numeric and in tertiles); smoking status (no, occasionally and daily); alcohol consumption (never, once or less per month, and more than once per month). In addition, the Alcohol Use Disorders Identification Test (AUDIT) was used (threshold set at 8 points), depression was assessed using the Patient Health Questionnaire, with a threshold set at 10 points 11, physical activity was assessed using the International Physical Activity Questionnaire, and blood pressure was measured three times (the average of the last two values was used) after a 5‐min resting period (OMRON HEM‐780, OMRON Healthcare, Lake Forest, IL, USA). Hypertension was defined as a blood pressure ≥140/90 mmHg or self‐reported physician diagnosis or currently receiving anti‐hypertensive medication. Data collection was conducted by trained field workers.

### Statistical analysis

Statistical analysis was conducted on stata 13.0 for Windows (StataCorp, College Station, TX, USA). Absolute and relative frequencies were used to describe categorical variables, which were compared using the chi‐squared test. Means and sd values were used to summarize numerical variables, which were compared against categorical variables with Student's *t*‐test. A regression model was fitted to study the association between Type 2 diabetes and ED; the Poisson family 12 and robust standard errors were specified. A crude and adjusted model were fitted, the latter accounting for age (numeric variable), assets index (numeric variable), BMI (numeric variable), smoking status, physical activity (numeric variable), alcohol consumption (AUDIT) and depression (raw score). These estimates are presented as prevalence ratios with 95% CIs. The area under the receiver‐operating characteristic curve (AUC) for each risk score was estimated with the *roctab* command, both overall and stratified by ED status.

### Ethics

All participants provided signed, informed consent, which, along with the study protocol and questionnaires, was approved by two institutional review boards: those of the Universidad Peruana Cayetano Heredia (Lima, Peru) and the London School of Hygiene and Tropical Medicine (London, UK).

## Results

### Study population

A total of 799 men with a mean (sd) age of 48.6 (10.7) years were included. The prevalence of Type 2 diabetes was 9.3% (95% CI 7.4–11.5); this represented 74 men, of whom 48.7% were not aware they had Type 2 diabetes. Further details of the study population are given in Table 1.

**Table 1:** Socio‐demographic and clinical characteristics of the study population according to diabetes status

	No diabetes	Diabetes	*P*
Age	*N*=724	*N*=74	<0.001
<40 years	29.1	9.5	
40–49 years	28.5	24.3	
50–59 years	23.6	44.6	
≥60 years	18.8	21.6	
Mean (sd) age, years	48.2 (10.7)	52.8 (9.2)	<0.001
Assets index	*N*=724	*N*=74	0.364
Low	31.8	25.7	
Middle	35.4	33.8	
Top	32.9	40.5	
Mean (sd)	245.5 (153.8)	294.9 (181.2)	0.010
Smoking status	*N*=724	*N*=74	0.639
Non‐smoker	74.2	78.4	
Occasional smoker	14.8	10.8	
Daily smoker	11.1	10.8	
Alcohol consumption	*N*=724	*N*=74	0.080
Never	20.0	31.1	
Once or less per month	60.9	54.1	
More than once per month	19.1	14.9	
Alcohol as per AUDIT	*N*=724	*N*=74	0.298
Negative	84.7	89.2	
Positive	15.3	10.8	
Physical activity	*N*=724	*N*=74	0.157
Low	23.9	33.8	
Moderate	31.6	29.7	
High	44.5	36.5	
Mean (sd)	5056.8 (6239.6)	4099.4 (5859.8)	0.207
BMI	*N*=724	*N*=74	0.069
<25 kg/m^2^	33.2	20.3	
25–29.9 kg/m^2^	45.0	51.4	
≥30 kg/m^2^	21.8	28.4	
Mean (sd)	27.0	28.1	<0.001
Hypertension	*N*=724	*N*=74	0.001
No	74.2	55.4	
Yes	25.8	44.6	
Mean (sd) systolic blood pressure	123.8 (14.4)	130.3 (19.9)	<0.001
Depression (PHQ‐9)	*N*=724	*N*=74	0.006
No	99.3	96.0	
Yes (score ≥10), *n*	0.7	4.1	
Erectile dysfunction	*N*=724	*N*=74	<0.001
No	93.9	75.7	
Yes	6.1	24.3	

AUDIT, Alcohol Use Disorders Identification Test; PHQ‐9, nine‐item Patient Health Questionnaire.

*P* values for categorical variables refer to the chi‐squared test, while for numerical variables they refer to Student's *t*‐test.

### Type 2 diabetes and erectile dysfunction

Overall, the proportion of men with ED was 7.8% (95% CI 6.1–9.8). There was an association between Type 2 diabetes and ED (*P*<0.001; Table 1). Moreover, ED was strongly associated with Type 2 diabetes in both unadjusted (prevalence ratio 3.82, 95% CI 2.40–6.07) and adjusted (prevalence ratio 2.71, 95% CI 1.57–4.66) regression models, signalling that ED occurrence in Type 2 diabetes is independent of other clinical characteristics, such as BMI and hypertension.

Men with ED did not have significantly higher postprandial glucose than men without ED (*P*=0.319): mean (sd) 6.7 (2.8) mmol/l vs 6.3 (2.3) mmol/l. A cross‐tabulation of impaired glucose tolerance according to oral glucose tolerance test and ED revealed a worse profile in men with ED (*P*<0.001): 59.7% were euglycaemic, 11.3% had impaired glucose tolerance and 29.0% had Type 2 diabetes; the respective rates for men without ED were 79.9%, 12.5% and 7.6%.

### Erectile dysfunction for undiagnosed Type 2 diabetes screening

**Figure 1:**
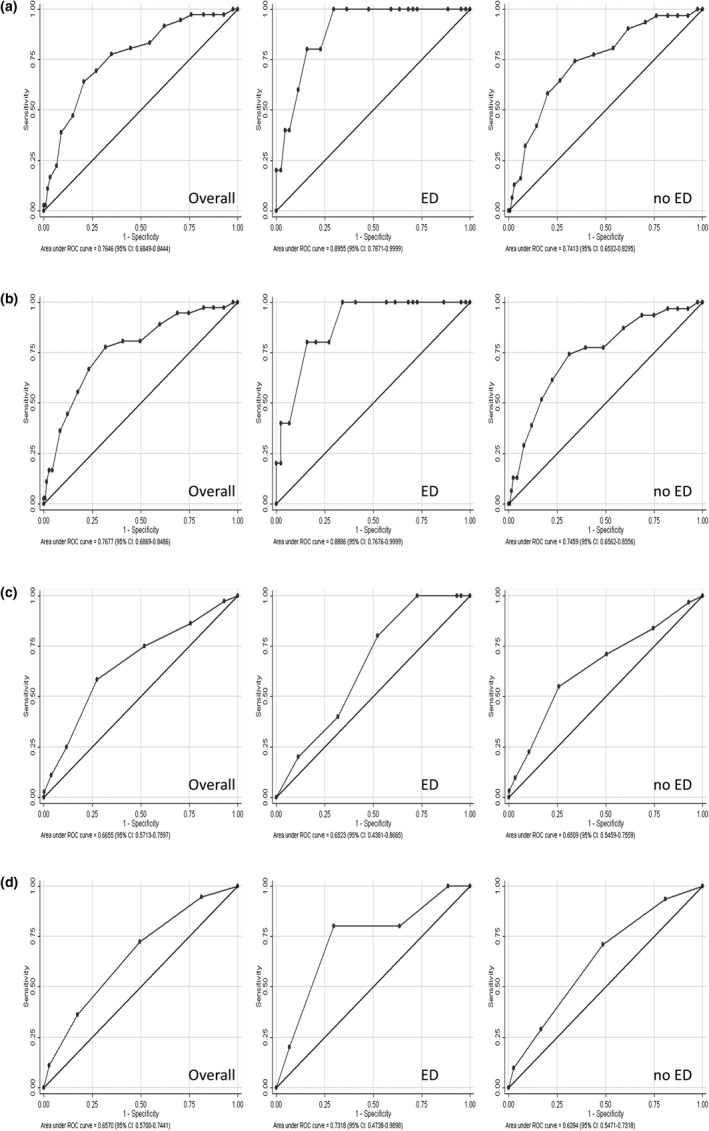
Area under the receiver‐operator curve (ROC; 95% CI) for each assessed diabetes risk score according to erectile dysfunction (ED) status: (a) LA‐FINDRISC, (b) FINDRISC, (c) American Diabetes Association (ADA) score and (d) Peruvian Risk Score.

Because the screening tools were designed for undiagnosed Type 2 diabetes, men who were aware they had Type 2 diabetes (*n*=38) were excluded from the following analysis. The mean scores using the four Type 2 diabetes risk screening tools assessed were 7.8 (FINDRISC), 8.1 (LA‐FINDRISC), 4.7 (ADA) and 1.5 (Peruvian Risk Score). All the risk scores, except the one based on ADA criteria, improved their discrimination accuracy (i.e. had a greater AUC) when they were applied to men with ED, in comparison to when they were applied to men without ED or overall (with and without ED together; Fig. 1). The largest increase was found in the LA‐FINDRISC, which had a 76.5% AUC overall, whilst this figure for men with ED reached up to 90.0% (Fig. 1a). This suggests that ascertaining the presence of ED before applying a Type 2 diabetes risk score could enhance the diagnostic accuracy of risk scores.

## Discussion

### Main findings

The prevalence of ED was higher in men with Type 2 diabetes than in otherwise healthy men. If the ascertainment of ED was carried out before applying well‐known risk scores for undiagnosed Type 2 diabetes, their diagnostic performance, based on AUC, would improve in men who reported ED, in relation to men who did not have this condition and overall. These findings suggest that screening for ED in men before screening for Type 2 diabetes could improve the chances of correctly identifying those at high risk of Type 2 diabetes.

### Results interpretation

Almost one‐quarter of men with Type 2 diabetes in the present study population had ED. This estimate was smaller than those reported in other studies 3. The explanation for this difference could lie in the definition of ED used in the present study; we based this on a single question whereas other studies used validated questionnaires to assess ED. In fact, it has been reported that different ED identification tools yield different prevalence estimates 3. Our results are conservative and warn of a higher prevalence of comorbid Type 2 diabetes with ED in Peru. In addition to the different instrument used to define ED, our study population was younger than that in many other studies addressing the association between Type 2 diabetes and ED; however, some studies with even younger populations have also reported a high prevalence of ED 3. This further supports the relevance of the role of ED ascertainment in assessing risk of Type 2 diabetes.

It has been reported that ED is a Type 2 diabetes‐associated factor 3, with even higher prevalence where metabolic control is not optimal 13. In men with Type 2 diabetes who are aware of their condition this could signal insufficient treatment or low adherence. Notwithstanding, in men unaware of having Type 2 diabetes, this could hide a long‐lasting illness. This is the most likely situation for the men in the present study, who did not undergo regular medical screening or have a high prevalence of Type 2 diabetes risk factors 6. Identifying men with ED (i.e. men with long‐lasting unknown Type 2 diabetes) could therefore improve the accuracy of Type 2 diabetes screening methods. Future studies need to prove, or disprove, this hypothesis in order for ED, a prevalent associated factor, to help in Type 2 diabetes screening and identification.

### Pathways between erectile dysfunction and diabetes

The association between Type 2 diabetes and ED has been extensively studied and summarized in systematic reviews pinpointing high ED prevalence in men with diabetes 3, 14. In addition to this epidemiological evidence, a strong case has been made to support the physiological pathways between these two conditions 15, 16, 17, 18. Although a comprehensive review of these pathways was beyond the scope of the present study, the possible mechanisms include: (1) hormonal deficiency (men with diabetes have lower testosterone levels); (2) endothelial dysfunction and dearth of nitric oxide at the penis circulation level, related to oxidative stress, advanced glycation end products and endothelins; and (3) impaired blood irrigation to the vasa nervorum at the penis (cavernous nerve) level.

The main strength of the present study is its assessment of the outcome variable based on an oral glucose tolerance test. The main limitation is the evaluation of the exposure variable based on only one question, whereas most studies have used validated questionnaires 3. If this non‐differential misclassification of the exposure of interest had had an effect on the results, then the point estimates of the regression model would have been towards the null. This was not the case because we reported strong associations even in the adjusted model. Not using a more comprehensive ED assessment tool could have prevented us from finding more cases for the stratified analysis; this could explain the wide CIs. Future studies should verify our results with larger sample size and using stronger methods to assess ED. Nevertheless, from a pragmatic point of view, our results suggest that, even with a simple question, assessment of ED could improve Type 2 diabetes screening at the population level.

In conclusion, ED is more common in men with Type 2 diabetes than in their otherwise healthy counterparts. It seems that ED screening, even with one simple question, before screening for Type 2 diabetes could enhance the odds of finding a true Type 2 diabetes case.

## Funding sources

Antonio Bernabe‐Ortiz is a Research Training Fellow in Public Health and Tropical Medicine (103994/Z/14/Z), funded by the Wellcome Trust.

## Competing interests

None declared.
